# Bee Venom Acupuncture Augments Anti-Inflammation in the Peripheral Organs of hSOD1^G93A^ Transgenic Mice

**DOI:** 10.3390/toxins7082835

**Published:** 2015-07-29

**Authors:** Sun-Hwa Lee, Sun-Mi Choi, Eun Jin Yang

**Affiliations:** 1 Division of Clinical Research, Korea Institute of Oriental Medicine, Daejeon 305-811, Korea; E-Mail: enddlsh@kiom.re.kr; 2 Executive Director of R&D, Korea Institute of Oriental Medicine, Daejeon 305-811, Korea; E-Mail: smchoi@kiom.re.kr

**Keywords:** amyotrophic lateral sclerosis (ALS), bee venom acupuncture (BVA), anti-inflammation

## Abstract

Amyotrophic lateral sclerosis (ALS) includes progressively degenerated motor neurons in the brainstem, motor cortex, and spinal cord. Recent reports demonstrate the dysfunction of multiple organs, including the lungs, spleen, and liver, in ALS animals and patients. Bee venom acupuncture (BVA) has been used for treating inflammatory diseases in Oriental Medicine. In a previous study, we demonstrated that BV prevented motor neuron death and increased anti-inflammation in the spinal cord of symptomatic hSOD1^G93A^ transgenic mice. In this study, we examined whether BVA’s effects depend on acupuncture point (ST36) in the organs, including the liver, spleen and kidney, of hSOD1^G93A^ transgenic mice. We found that BV treatment at ST36 reduces inflammation in the liver, spleen, and kidney compared with saline-treatment at ST36 and BV injected intraperitoneally in symptomatic hSOD1^G93A^ transgenic mice. Those findings suggest that BV treatment combined with acupuncture stimulation is more effective at reducing inflammation and increasing immune responses compared with only BV treatment, at least in an ALS animal model.

## 1. Introduction

Amyotrophic lateral sclerosis (ALS) is characterized by progressive degeneration of motor neurons and muscle weakness. The death of ALS patients is caused by respiratory failure within 3–5 years of the diagnosis. ALS has two types: familial ALS (fALS) caused by genetic mutations, including superoxidase dismutase 1 (SOD1), alsin, senataxin, angiogenin, VAMP-associated protein B, dynactin, transactive response (TAR) DNA-binding protein 43 (TDP43), fused in sarcoma (FUS) and C9ORF72; and sporadic ALS (sALS), which includes ninety percent of all ALS cases and is induced by various environmental and genetic factors. The etiology of ALS is varied and there is no effective therapy for ALS patients. Riluzole, a glutamate release inhibitor approved by the FDA, is used only as a medical treatment for expansion of life by 3–5 months [1]. 

Bee venom (BV) is used for anti-inflammatory, anti-nociceptive, and anti-allergic effects in allergic rhinitis mice, complete Freund’s adjuvant (CFA)-induced arthritis models, and neuropathic pain models [2,3,4]. In addition, BV treatment prevents the loss of dopaminergic neurons in 1-methyl-4-phenyl-1,2,3,6-tetrahydropyridine (MPTP)-induced Parkinson’s disease (PD) and motor neurons in hSOD1^G93A^-overexpressed ALS-mimic transgenic mice [5,6,7]. However, it is unclear whether BV’s effects depend on acupuncture points or not. Therefore, the purpose of this study is to investigate whether BV treatment at ST36 is more effective than only BV treatment for the reduction of inflammation in the peripheral organs, including the liver, spleen, and kidney, in symptomatic hSOD1^G93A^ transgenic mice. 

In this study, we found that BV treatment at ST36 reduced inflammation in the liver, spleen, and kidney compared with that of symptomatic hSOD1^G93A^ transgenic mice treated with saline at ST36 and those injected with BV intraperitoneally. Those findings suggest that BV treatment combined with acupuncture stimulation is more effective at reducing inflammation and increasing immune responses than is BV-only treatment, at least in an ALS animal model.

## 2. Results

### 2.1. BV Treatment at ST36 Reduces Inflammatory Proteins in the Liver of hSOD1^G93A^ Transgenic Mice

**Figure 1:**
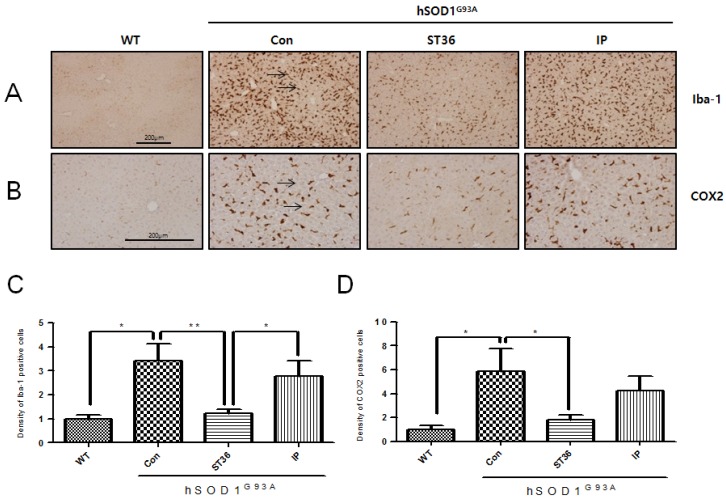
The effects of BV on inflammation in the liver of hSOD1^G93A^ mice. BV treatment at ST36 reduces inflammation in the liver in hSOD1^G93A^ mice. Immunohistochemical staining of paraffin-embedded sections of non-Tg mice (WT, *n* = 3), hSOD1^G93A^ mice (Con, *n* = 3), BV-treatment at Joksamli (ST36) acupuncture point in hSOD1^G93A^ mice (ST36, *n* = 7) and BV-treatment intraperitoneally in hSOD1^G93A^ mice (IP, *n* = 4). Representative images of immunohistochemistry with Iba-1 (**A**) and COX2 (**B**) in the liver of hSOD1^G93A^ mice or non-Tg mice. Quantification of Iba-1 (**C**) and COX2 (**D**) immunoreactivity (IR). It assigned the optical density of WT to one and analyzed relative optical density of Con, ST36, and IP. Data are expressed as the mean ± SEM. * *p* < 0.05, ** *p* < 0.01 from a one-way ANOVA with a Newman-Keuls test. Scale bar indicates 200 μm. WT: non-Tg; Con: saline-treatment at ST36; ST36: BV-treatment at ST36; IP: BV-injection intraperitoneally.

To investigate the effects of BV on inflammation specific to injection method, we conducted BV treatment two ways, namely at acupuncture point ST36 and intraperitoneally (i.p.), in 14-week-old hSOD1^G93A^ transgenic mice. As shown in Figure 1A, the expression level of Iba-1 in hepatocytes of the liver of hSOD1^G93A^ transgenic mice was increased by 3.4-fold compared with age-matched wild type (WT) (1 ± 0.24) mice. Furthermore, we found that BV treatment at ST36 significantly reduced Iba-1 by 2.8-fold compared with age-matched hSOD1^G93A^ transgenic mice. In addition, the anti-inflammatory effects of BV treatment at ST36 increased by 2.3-fold compared with BV injected by i.p. in the hSOD1^G93A^ mice. To confirm the anti-inflammatory effects of BV treatment at ST36, we studied the expression level of the cyclooxygenase 2 (COX2) protein in the liver of WT and hSOD1^G93A^ transgenic mice. COX2 positive hepatocytes were increased in the liver of hSOD1^G93A^ transgenic mice compared with WT (1 ± 0.55) mice, but BV treatment at ST36 significantly reduced them by 3.3-fold compared with saline-treated hSOD1^G93A^ transgenic mice. Those findings suggest that BV treatment at ST36 is effective at reducing inflammation in the liver of hSOD1^G93A^ transgenic mice. 

### 2.2. BV Treatment at ST36 Reduces Inflammation in the Spleen of hSOD1^G93A^ Transgenic Mice

**Figure 2:**
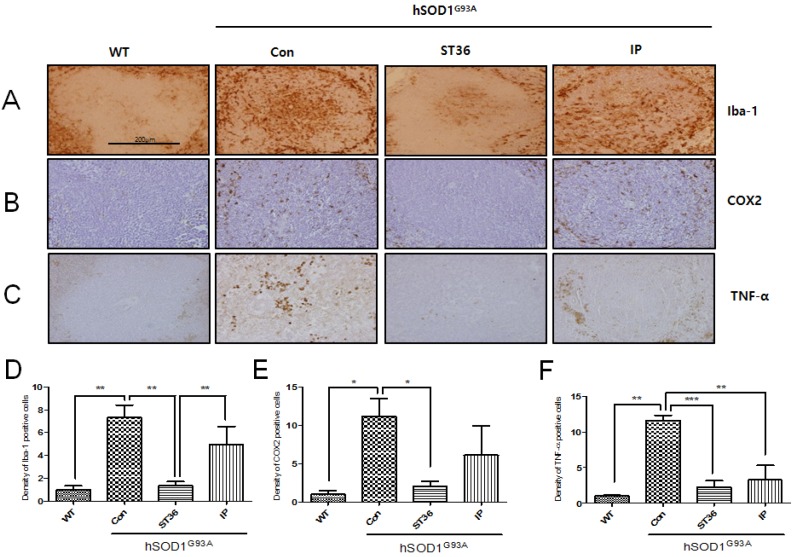
The effects of BV in the spleen of hSOD1^G93A^ mice. BV treatment decreases the expression of inflammatory proteins in the spleen of hSOD1^G93A^ mice. Representative images of the immunohistochemistry of inflammation-related proteins Iba-1 (**A**), COX2 (**B**), and TNF-α (**C**) in the spleen of three groups (Con, ST36, and IP) of hSOD1^G93A^ mice and WT mice. Quantification of Iba-1 (**D**), COX2 (**E**) and TNF-α (**F**) IR. It assigned the optical density of WT to 1 and analyzed relative optical density of Con, ST36, and IP. Data are shown as the mean ± SEM. * *p* < 0.05, ** *p* < 0.01, *** *p* < 0.001 from a one-way ANOVA with a Newman-Keuls test. Scale bar indicates 200 μm. WT: non-Tg; Con: saline-treatment at ST36; ST36: BV-treatment at ST36; IP: BV-injection intraperitoneally.

To study the anti-inflammatory effects of BV treatment at ST36 in the spleen, which is involved in the immune response, we immunostained with Iba-1, COX2, and tumor necrosis factor (TNF)-α antibodies for the tissues of WT or hSOD1^G93A^ transgenic mice. As shown in Figure 2A, we found that the expression level of Iba-1 was greatly increased by 7.3-fold in the white pulp of the spleen of the hSOD1^G93A^ transgenic mice compared with WT (1 ± 0.59) mice. BV treatment at ST36 significantly reduced Iba-1 by 5.2-fold compared with saline-treated hSOD1^G93A^ transgenic mice. In addition, the expression level of Iba-1 in the white pulp of the spleen was decreased by 3.5-fold by BV treatment at ST36 compared with BV injected i.p. in the hSOD1^G93A^ transgenic mice. BV treatment at ST36 in hSOD1^G93A^ transgenic mice also significantly reduced COX2-immuno positive cells by 5.6-fold, which were increased by 11.2-fold compared with WT mice (1 ± 0.86). As a pro-inflammatory protein, TNF-α expression in the white pulp of the spleen of the hSOD1^G93A^ transgenic mice also increased by 11.7-fold compared with WT mice (1 ± 0.25), but BV treatment at ST36 significantly reduced its expression by 5.3-fold compared with hSOD1^G93A^ transgenic mice. BV injection by i.p. decreased the expression of TNF-α by 3.5-fold in the spleen of the hSOD1^G93A^ transgenic mice compared with saline-treated hSOD1^G93A^ transgenic mice. BV treatment at ST36 seems to be more effective at reducing the expression of the pro-inflammatory protein TNF-α in the spleen than BV-injected i.p., but it was not significantly different. These findings suggest that BV treatment at ST36 could augment immune responses by reducing the inflammatory proteins in the spleen of the hSOD1^G93A^ transgenic mice. 

**Figure 3:**
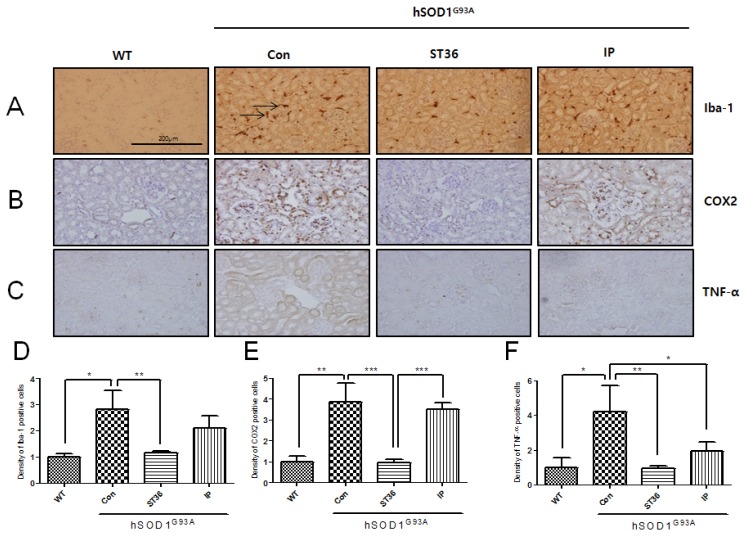
The effects of BV in the kidney of hSOD1^G93A^ mice. BV treatment at ST36 is more effective at reducing the expression of inflammatory proteins in the kidney of the hSOD1^G93A^ mice. Representative images of kidney tissue immunostained with Iba-1 (**A**), COX2 (**B**), and TNF-α (**C**) of three groups of hSOD1^G93A^ mice and WT mice. Quantification of immune-positive cells with Iba-1 (**D**), COX2 (**E**) and TNF-α (**F**) IR. It assigned the optical density of WT to 1 and analyzed relative optical density of Con, ST36, and IP. Data are shown as the mean ± SEM. * *p* < 0.05, ** *p* < 0.01, *** *p* < 0.001 from a one-way ANOVA with a Newman-Keuls test. Scale bar indicates 200 μm. WT: non-Tg; Con: saline-treatment at ST36; ST36: BV-treatment at ST36; IP: BV-injection intraperitoneally.

### 2.3. BV Treatment at ST36 Downregulates Inflammation in the Kidney of hSOD1^G93A^ Transgenic Mice

We examined the expression level of inflammatory proteins and the effects of BV in the kidney of the hSOD1^G93A^ transgenic mice. The expression of Iba-1 was increased by 2.8-fold in the kidney, as was shown in the liver and spleen, in the hSOD1^G93A^ transgenic mice compared with age-matched WT mice (1 ± 0.23) (Figure 3A). BV treatment at ST36 in the kidney of hSOD1^G93A^ transgenic mice reduced its expression by 2.3-fold compared with saline-treated hSOD1^G93A^ transgenic mice (Figure 3A). COX2 protein increased by 3.8-fold in the renal tubules and renal glomeruli of the kidney in symptomatic hSOD1^G93A^ transgenic mice compared with age-matched WT mice (1 ± 0.44) (Figure 3B). BV treatment at ST36 reduced COX2 expression in the kidney by 4.2-fold compared with saline-treated hSOD1^G93A^ transgenic mice. Furthermore, BV treatment at ST36 is more effective, by 4.2-fold, at decreasing COX2 expression compared with i.p. injection of BV in the hSOD1^G93A^ transgenic mice. TNF-α positive cells were increased by 4.2-fold in the renal tube of the kidney of hSOD1^G93A^ transgenic mice compared with WT mice (1 ± 0.93) (Figure 3C). However, BV treatment at ST36 significantly reduced, by 4.7-fold, TNF-α cytoplasmic staining cells in the renal tube of the kidney compared with saline-treatment at ST36 in hSOD1^G93A^ transgenic mice. BV injection i.p. in hSOD1^G93A^ transgenic mice also reduced, by 2.2-fold, the TNF-α expression in the kidney. BV treatment at ST36 showed more reduction of the expression of TNF-α in the renal tube of the kidney compared with that of i.p. injection of BV in hSOD1^G93A^ transgenic mice, but this difference was not significant. Those findings suggest that BV treatment at ST36 could improve kidney function by increasing the anti-inflammatory proteins in an ALS animal model.

## 3. Discussion

ALS causes the loss of motor neurons in the brainstem, cerebral cortex, and spinal cord and leads to irreversible paralysis of muscles and finally to respiratory impairment. There are several cellular and molecular pathological mechanisms involved, such as glutamate excitation, inflammatory events, oxidative stress, mitochondrial dysfunction, protein aggregation, and energy failure, as in other neurodegenerative diseases. However, it is insufficient to develop treatments and preventative measures for these biomarkers of ALS. Neuroinflammation in the central nervous system (CNS) from ALS contributes to the disease process and the immune system of sALS patients is altered by immune cells, including remarkable reductions in CD4+CD25+ T-regulatory (T-reg) cells as well as CD14+ monocytes [8]. This suggests that the reduction of T-reg cells in the blood affects the CNS immune system by involving activated microglia in ALS degeneration [9]. Several papers have reported anti-inflammatory therapy using Copaxone, Cyclosporine, and minocycline in animal models and clinical trials, but those have limitations for the treatment of ALS [10]. In a previous study, we demonstrated immune dysfunction of organs, including the lungs and spleen, in hSOD1^G93A^ transgenic mice and that electroacupuncture and melittin treatment enhanced anti-inflammation proteins [11,12]. Based on previous data, we investigated the effects of BV on inflammation of organs, including the spleen, liver, and kidney, of symptomatic hSOD1^G93A^ transgenic mice. We found that BV treatment at ST36 reduced inflammatory proteins, including Iba-1, COX2, and TNF-α, in the liver, spleen, and kidney of hSOD1^G93A^ transgenic mice compared with BV injected by i.p. This suggests that BV’s effects may be more effective with treatment at an acupuncture point and it explains the synergistic effect of acupuncture combined with BV compared with BV treatment only.

Finkelstein *et al.* reported liver abnormalities and atrophy, and an increase of cytokines and hepatic lymphocytes in hSOD1^G93A^ transgenic mice [13]. In our study, we found an increase of Iba-1 and COX2 positive cells in the liver of symptomatic hSOD1^G93A^ transgenic mice, but BV treatment at ST36 significantly reduced the expression level of inflammatory proteins, including Iba-1 and COX2 compared with BV injected i.p. in symptomatic hSOD1^G93A^ transgenic mice. This suggests that BV treatment at the acupuncture point ST36 may reduce hepatotoxicity from inflammation and affect liver metabolism in ALS patients.

In hSOD1^G93A^ mice as an ALS animal model, the spleen is markedly reduced in size and weight compared with age-matched B6 wild type mice even though their spleen cell number is identical. In addition, splenic follicular architecture, T cell function, and the lymphoproliferative response are decreased in end-stage hSOD1^G93A^ transgenic mice. Immune dysregulation affecting both the adaptive and innate immune systems is a consistent hallmark in ALS [14]. In our study, we observed that the white pulp, the immune-related component of the spleen, was strongly immunostained with anti-Iba-1, anti-COX2, and anti-TNF-α in symptomatic hSOD1^G93A^ transgenic mice compared with age-matched non-transgenic mice. Furthermore, BV treatment at ST36 reduced the expression level of Iba-1, COX2, and TNF-α compared with saline-treatment at ST36 in symptomatic hSOD1^G93A^ transgenic mice. Those findings suggest that BV treatment at ST36 improves the immune regulation of hSOD1^G93A^ transgenic mice through a synergic effect of acupuncture combined with BV. 

Jonsson *et al.* have reported that granular inclusion of mutant SOD1 protein is detected in the liver and kidney by immunohistochemical analysis of ALS patients [15]. We observed increased expression of inflammation-related proteins, including Iba-1, COX2, and TNF-α, in the renal tubules or renal glomeruli in symptomatic hSOD1^G93A^ transgenic mice. BV treatment at ST36 significantly increased anti-inflammation proteins in the kidney compared with saline-treatment at ST36 in hSOD1^G93A^ transgenic mice. This suggests that BV treatment at this acupuncture point could improve kidney dysfunction in an ALS animal model. In addition, we suggest that the mechanism of BV treatment at ST36 may occur by combined effect of some components of BV suppressing inflammatory signaling and the activation of the endogenous modulatory systems by acupoint stimulation. Therefore, it should be required to confirm the activity of individual components of BV. Future studies should investigate whether BV’s effects at ST36 are specific or generalized to other acupuncture points. In addition, the relationship between acupuncture point stimulation and meridian, and the mechanism of BV’s synergistic effects when combined with acupuncture compared with BV alone need to be explored. 

## 4. Materials and Methods

### 4.1. Animals

All mice were handled in accordance with the United States National Institutes of Health guidelines, and all procedures were approved by the Institutional Animal Care and Use Committees of the Korea Institute of Oriental Medicine (Protocol number: #13-109). Hemizygous transgenic B6SJL mice carrying the mutant human SOD1 gene, which has a glycine-to-alanine base pair mutation at the 93^rd^ codon of the cytosolic Cu/Zn superoxide dismutase (hSOD1^G93A^), were originally obtained from Jackson Laboratory (Bar Harbor, ME, USA). 

Transgenic mice were identified using polymerase chain reaction (PCR) as described previously [16]. All of the mice were kept in standard housing with free access to water and standard rodent chow purchased from Orient Bio (Orient, Seongnam-si, Gyeonggi-do, Korea).

### 4.2. Bee Venom Treatment

Bee venom (BV) was purchased from Sigma (St. Louis, MO, USA) and diluted with saline. At a dose of 0.1 µg/g, bee venom was injected bilaterally at the Joksamli (ST36) acupuncture point (*n* = 7) or intraperitoneally (IP; *n* = 4) in 14 week-old hSOD1^G93A^ transgenic mice. The mice were treated with BV once every other day for two weeks. According to the human acupuncture point landmark and a mouse anatomical reference [17], the ST36 acupuncture point is anatomically located at 5 mm below and lateral to the anterior tubercle of the tibia. Non-transgenic (WT; *n* = 3) and transgenic hSOD1^G93A^ mice (Con; *n* = 3) were injected at the ST36 acupuncture point with normal saline of an equal volume. 

### 4.3. Tissue Preparation and Immunohistochemistry

hSOD1^G93A^ mice were anesthetized with pentobarbital and perfused with phosphate-buffered saline (PBS). The liver, spleen and kidney were removed and fixed in 4% paraformaldehyde for three days at 4 °C After three days, the liver, spleen and kidney were embedded in paraffin. The tissues were 5 µm-thick sections and were mounted on glass slides. The tissue sections were prepared for immunostaining through xylene treatment and gradual rehydration with 95%–75% ethanol. Following de-paraffinization, the slides were treated with 3% hydrogen peroxide (H_2_O_2_) for 15 min to inactivate endogenous peroxidases and then blocked in 5% bovine serum albumin (BSA) in 0.01% PBS-Triton X–100 (Sigma-Aldrich, Oakville, ON, Canada) for 1 h at room temperature. The sections were then incubated with various primary antibodies, including anti-Iba-1 (Wako, Osaka, Japan), anti-TNF-α (Abcam, Cambridge, UK), and anti-COX2 (Epitomics, Burlingame, CA, USA), overnight. Next, the sections were incubated with the secondary antibody for 1 h. For visualizing, the ABC kit and 3,3'-diaminobenzidine (DAB)/H_2_O_2_ substrate were used with a hematoxylin counterstain. After rinsing, the sections were dehydrated in ethanol, cleared in xylene, and coverslipped. Immunostained tissues were observed with a light microscope (Olympus, Tokyo, Japan) and analyzed by Image J 1.46j software (NIH) (GraphPad Software, San Diego, CA, USA).

### 4.4. Statistical Analysis

All data were analyzed using GraphPad Prism 5.0 (GraphPad Software, San Diego, CA, USA) and are presented as the mean ± standard error of the mean (SEM) where indicated. The results of immunohistochemistry and Western blots were analyzed using one-way ANOVAs followed by Newman-Keuls tests. Statistical significance was set at *p* < 0.05.

## 5. Conclusions

In summary, we examined whether BVA’s effects depend on acupuncture point (ST36) in the organs, including the liver, spleen and kidney, of hSOD1^G93A^ transgenic mice. We found that BV treatment at ST36 reduces inflammation-related proteins including Iba-1, COX2, TNF-α in the liver, spleen, and kidney compared with saline-treatment at ST36 and BV injected i.p. in symptomatic hSOD1^G93A^ transgenic mice. Those findings suggest that BV treatment combined with acupuncture stimulation is more effective at reducing inflammation and increasing immune responses compared with only BV treatment, at least in an ALS animal model. 
